# Serological Distribution of *Salmonella enterica* subsp. Isolated from Feces of Domesticated Crested Gecko (*Correlophus ciliates*) in Busan Province, South Korea

**DOI:** 10.3390/life15030405

**Published:** 2025-03-05

**Authors:** Il Kwon Bae, Yon-koung Park, So Hyun Park, Jun Sung Hong

**Affiliations:** 1 Department of Companion Animal Health and Sciences, Silla University, Busan 46958, Republic of Korea; ikbae@silla.ac.kr; 2 Microbiology Division, Busan Metropolitan City Institute of Health and Environment, Busan 46616, Republic of Korea; akaciaa@naver.com (Y.-k.P.); sohyuny0711@naver.com (S.H.P.)

**Keywords:** crested gecko, *Salmonella*, serotype, *Citrobacter*, enteric bacteria, AmpC

## Abstract

Geckos are often considered to be reservoirs of zoonotic pathogens. This study was conducted to describe the prevalence and characteristics of pathogens isolated from fecal samples of crested geckos in South Korea. A total of 76 fecal samples were collected from 76 domesticated crested geckos in independent captivity. To determine bacterial profiles, matrix-assisted laser desorption ionization-time of flight mass spectrometry (MALDI-TOF MS), the disk diffusion method, PCR and direct sequencing, and the Kauffmann–White scheme for serotyping *Salmonella* species were performed. A total of 107 Gram-negative isolates were identified as belonging to 50 *Citrobacter* species, 33 *Salmonella* enterica subsp., 8 *Serratia marcescens*, 8 *Klebsiella* species, 3 *Morganella morganii*, 2 *Enterobacter cloacae*, 2 *Pseudomonas aeruginosa*, and 1 *Acinetobacter* species. Most of the isolates were susceptible to antibiotics tested in this study. The chloramphenicol acetyltransferase (cat) gene was detected in one *M. morganii* isolate, and the class C beta-lactamase (AZECL-14) gene was detected in one *E. cloacae*. The most prevalent somatic (O) antigens of the groups were C (n = 23) and D (n = 7), and 8 different serotypes were identified among the 33 *Salmonella* enterica subsp. isolates. Five of eight *Salmonella* serotypes have not been previously reported among clinical isolates in South Korea. Our results reveal that enteric bacteria have not been shared between crested geckos and humans, at least in South Korea.

## 1. Introduction

Lizards are known to be the most common reptiles, with more than 7000 species, and are found worldwide, except in Antarctica [1]. Geckos belonging to the Gekkonidae family are some of the best-domesticated lizard species for private keepers to breed because their diet is relatively simple in captive care [2,3]. Among them, the crested gecko, *Correlophus ciliates*, has recently become one of the most popular species of gecko by household breeders in the pet trade market worldwide [4].

Lizards are often considered carriers or reservoirs of zoonotic pathogens [5,6]. In this regard, non-typhoidal *Salmonella*, *Citrobacter* species, *Enterobacter* species, *Escherichia coli*, *Serratia marcescens*, and *Klebsiella* species have been isolated from various species of gecko in many reports [5,6,7,8]. These organisms in the intestinal tract of the geckos cannot cause zoonotic diseases to themselves [9]. However, they can potentially infect other animals and immunocompromised humans as acute pathogens through direct or indirect transmission. In the case of captive care, humans can be infected directly by close contact and handling with the gecko [10]. In addition, humans may become infected indirectly by manipulating certain environments that are contaminated by zoonotic pathogens, such as feces or the fecal droppings (excreta) of geckos [11].

Among zoonotic pathogens, *Salmonella enterica* is well known as pathogenic bacteria that cause diseases in both animals and humans worldwide [12,13]. For the classification of *S. enterica*, serogrouping and serotyping identification via the White–Kauffmann–Le Minor scheme are primarily used as gold-standard tools worldwide [14]. To date, non-typhoidal *Salmonella* species have been identified according to their serogroup and serotype and divided into six serogroups (B, C1, C2, D, E, and other serogroups) that comprise more than 2600 serotypes based on the distinct combination of *Salmonella* O and H antigens [15,16]. *Citrobacter* species also colonize animal and human intestines as opportunistic pathogens [17]. At present, the genus *Citrobacter* includes 18 species [18], which often produce regulated chromosomal Ambler class C β-lactamases (AmpCs), which confer resistance to second-generation cephalosporins such as cefoxitin [19,20,21].

The aim of this study was to describe the prevalence and characteristics of zoonotic Gram-negative pathogens, especially *Salmonella enterica* subspecies, isolated from fecal samples of crested geckos in South Korea.

## 2. Materials and Methods

### 2.1. Sample Collection and Study Design

In this study, a total of 76 fecal samples using sterile cotton swabs were collected from 76 domesticated crested geckos obtained by a private breeder in a household in Busan province in 2023. These 76 crested geckos were bred and reared in independent captivity without other human or animal contact before, during, or after collection. Fecal samples (n = 76) of captive crested geckos included 20 samples (categorized as group 1) from crested geckos fed superworms (*Zophobas morio* and *Tenebrio molitor*) and 56 samples (categorized as group 2) from crested geckos fed crickets (*Gryllidae*) (Table S1).

No crested geckos received antimicrobial treatments within the 3 months prior to sampling, and all geckos were in clinically healthy condition. Fecal samples in transport medium (COPAN DIAGNOSTICS, Murrieta, CA, USA) were delivered to the laboratory within 6 h of collection. All fecal samples were incubated at 37 °C for 24 h in Brain Heart Infusion broth (Kisan Bio, Seoul, Republic of Korea). After full incubation, we streaked on MacConkey agar (Duksan Science, Seoul, Republic of Korea).

### 2.2. Identification of Bacteria

The identification of Gram-negative isolates positively cultured on MacConkey agar was confirmed by matrix-assisted laser desorption ionization-time of flight mass spectrometry (MALDI-TOF MS) with a Vitek-MS (bioMérieux, Marcy-l’Etoile, France) using a single colony. MALDI-TOF MS is a mass spectrometry method for accurate identification of bacteria. This method is based on the analysis of ribosomal proteins of bacteria in the mass range of 2000 to 20,000 Daltons. These proteins are ionized into charged molecules to measure the mass-to-charge (*m*/*z*) ratio. When the matrix (energy-absorbing solution) crystallizes upon drying, the sample trapped inside also cocrystallizes. A laser ionizes the sample, generating singly protonated ions. Based on the time of flight for each protonated ion, a characteristic mass spectrum is generated. This spectrum with peaks specific to genus and species unique to individual types of bacteria is compared with a database. The protocol is as follows: (i) smear a fresh single colony onto a polished steel target plate, (ii) overlay with 1 uL of a matrix solution, and (iii) air dry at room temperature.

### 2.3. Antimicrobial Susceptibility Testing (AST)

The disk diffusion method was used to assess the antimicrobial susceptibility phenotypes of all the isolates. The purpose of the disk diffusion method is to determine the susceptibility to various antimicrobial compounds. When a 6 mm filter paper disk impregnated with a known concentration of an antibiotic is placed on a Mueller–Hinton agar inoculated with a suspension of bacteria. After 37 °C for 24 h incubation, the antimicrobial begins to diffuse into the surrounding agar depending on the sensitivity or resistance of bacteria to antibiotics. The susceptibility of *Enterobacteriaceae* was evaluated using a 6 mm disks (Oxoid Ltd., Basingstoke, UK) for ampicillin (10 ug), piperacillin (20 ug), cefotaxime (5 ug), ceftazidime (10 ug), cefoxitin (30 ug), amikacin (30 ug), streptomycin (10 ug), chloramphenicol (30 ug), tetracycline (30 ug), ciprofloxacin (10 ug), aztreonam (30 ug), imipenem (10 ug), and cotrimoxazole (25 ug). The susceptibility profiles of *Salmonella* species to the following antibiotics were evaluated for ampicillin, cefazolin (30 ug), cefotetan (30 ug), ceftazidime, cefotaxime, cefoxitin, imipenem, amikacin, gentamicin (10 ug), streptomycin, ciprofloxacin, tetracycline, aztreonam, chloramphenicol, and cotrimoxazole. The susceptibility of *Acinetobacter* species was evaluated for piperacillin, cefotaxime, ceftazidime, amikacin, ciprofloxacin, cotrimoxazole, and imipenem. The susceptibility of *P. aeruginosa* was evaluated for piperacillin, ceftazidime, amikacin, ciprofloxacin, aztreonam, and imipenem.

The quality control strains *E. coli* American-type culture collection (ATCC) 25922 and *P. aeruginosa* ATCC 27853 were utilized. The results were interpreted according to the Clinical and Laboratory Standards Institute guidelines (CLSI M100-S32, 2023).

### 2.4. Serogrouping and Serotyping of Salmonella Species

Serotyping of *Salmonella* species isolates was carried out according to the Kauffmann–White scheme [14]. The somatic (O) antigens of each *Salmonella* species isolate were determined using the slide agglutination method with *Salmonella* polyvalent and monovalent somatic (O) antisera (S&A Reagents Lab Ltd., Bangkok, Thailand). This scheme differentiates isolates by determining the saccharidic component of the lipopolysaccharide exposed on the bacteria’s surface. Additional serotyping was performed with *Salmonella* H antiserum (Becton, Dickinson and Company, Sparks, MD, USA) using the tube agglutination method. The results of O and H antigen agglutination for each *Salmonella* species isolate were combined, and the specific serotype was confirmed according to the antigenic formulae of *Salmonella* serovars [22].

### 2.5. Detection of Antimicrobial Resistance Genes

The template DNA was extracted with a CicaGeneus Total DNA Prep Kit (KANTO Chemical, Tokyo, Japan). *Top* DNA polymerase, dNTP, 1.5 mM MgCl_2_, stabilizer/tracking dye, template DNA, and primers were used for PCR reactions. DNA sequencing of antimicrobial resistance (AMR) genes, including common plasmid-mediated AmpC β-lactamase (MOX-type, CMY-type, LAT-type, BIL-type, DHA-type, ACC-type, MIR-type, ACT-type, and FOX-type) genes and the chloramphenicol acetyltransferase (CAT) gene, were performed for cefoxitin- or chloramphenicol-non-susceptible isolates using PURE^TM^ PCR Cleanup Kit (INFUSION TECH, Seoul, Republic of Korea), respectively. The PCR program consisted of a single cycle of 5 min at 94 °C, followed by 35 cycles of 30 s at 94 °C, 30 s at 55 °C (CAT gene) or 60 °C (AmpC β-lactamase), and 30 s at 72 °C, followed by a final extension at 72 °C for 5 min. The sequences were compared with published DNA sequences using the BLAST network service. The primers used in this study are listed in Table 1.

## 3. Results

### 3.1. The Prevalence of Gram-Negative Bacteria

Among 76 fecal samples collected from 76 captive crested geckos domesticated in independent captivity, a total of 107 Gram-negative isolates were positively cultured and identified, including 37 *Citrobacter freundii* (34.6%, 37/107), 33 *Salmonella* species (30.8%), 8 *Serratia marcescens* (7.5%), 6 *Citrobacter amalonaticus* (5.6%), 6 *Klebsiella oxytoca* (5.6%), 5 *Citrobacter youngae* (4.7%), 3 *Morganella morganii* (2.8%), 2 *Citrobacter murliniae* (1.9%), 2 *Enterobacter cloacae* (1.9%), 2 *Pseudomonas aeruginosa* (1.9%), 2 *Klebsiella pneumoniae* (1.9%), and one *Acinetobacter guillouiae* (0.9%). The 107 Gram-negative isolates collected from each of the 76 domesticated crested geckos are listed in Table S1.

The isolation of Gram-negative bacteria from groups 1 and 2, categorized according to diet type, yielded different results. In group 1, for the crested geckos fed superworms, 32 isolates from fecal samples from each of the 16 crested geckos were identified. Among them, *C. freundii* (n = 10) was the most prevalent, followed by *S. marcescens* (n = 8), *C. amlonaticus* (n = 6), *C. murliniae* (n = 2), *C. youngae* (n = 1), *K. oxytoca* (n = 1), *M. morganii* (n = 1), *A. guillouiae* (n = 1), *P. aeruginosa* (n = 1), and *Salmonella* species (n = 1).

On the other hand, in group 2, for crested geckos fed crickets, 75 isolates from fecal samples from each of the 41 crested geckos were identified. *Salmonella* species (n = 32) were dominant, followed by *C. freundii* (n = 27), *Klebsiella oxytoca* (n = 5), *C. youngae* (n = 4), *M. morganii* (n = 2), *K. pneumoniae* (n = 2), *E. cloacae* (n = 2), and *P. aeruginosa* (n = 1).

### 3.2. Antimicrobial Resistance

**Figure 1:**
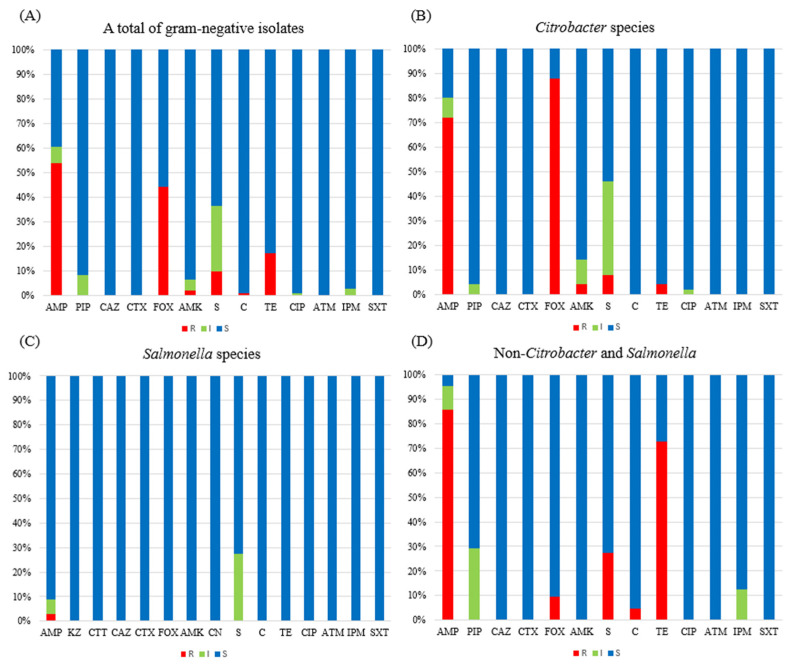
Antimicrobial susceptibility of 107 Gram-negative isolates tested in this study: (**A**) A total of Gram-negative isolates (n = 107). (**B**) *Citrobacter* species (n = 50). (**C**) *Salmonella* species (n = 33). (**D**) Non-*Citrobacter* and *Salmonella* isolates (n = 24). Abbreviation: S, susceptible; I, intermediate; R, resistant; AMP, ampicillin; PIP, piperacillin; KZ, cefazolin; CTT, cefotetan; CAZ, ceftazidime; CTX, cefotaxime; FOX, cefoxitin; AMK, amikacin; CN, gentamicin; S, streptomycin; C, chloramphenicol; TE, tetracycline, CIP, ciprofloxacin; ATM, aztreonam; IPM, imipenem; SXT, cotrimoxazole.

Figure 1 presents the AMR profiles of the 107 Gram-negative bacteria isolated from the fecal samples of the crested geckos in this study. The strains were phenotypically non-susceptible, including resistance and intermediate phenotypes, to ampicillin (63/104, 60.6%), piperacillin (9/107, 8.4%), cefoxitin (46/104, 44.2%), amikacin (7/107, 6.5%), streptomycin (38/107, 36.5%), chloramphenicol (1/104, 1.0%), tetracycline (18/104, 17.3%), ciprofloxacin (1/107, 0.9%), and imipenem (3/107, 2.8%), but they were susceptible to ceftazidime, cefotaxime, aztreonam, and cotrimoxazole.

Among the 50 *Citrobacter* species, the highest rate of non-susceptibility was 88.0% (n = 44) for cefoxitin, followed by 80.0% for ampicillin, 46.0% for streptomycin, 14.0% for amikacin, 4.0% for piperacillin, 4.0% for tetracycline, and 2.0% for ciprofloxacin, with susceptibility to ceftazidime, cefotaxime, chloramphenicol, aztreonam, imipenem, and cotrimoxazole. In addition, all six *C. amalonaticus* isolates showed the same AMR phenotype.

A total of 33 *Salmonella* species (*Salmonella enterica* subsp.) were non-susceptible to ampicillin (n = 3, 9.1%) and streptomycin (n = 9, 27.3%) and were susceptible to cefazolin, cefotetan, ceftazidime, cefotaxime, cefoxitin, amikacin, gentamicin, chloramphenicol, tetracycline, ciprofloxacin, aztreonam, imipenem, and cotrimoxazole. The AMR profiles were not significantly characterized by *Salmonella* serotypes (Table 2).

In 24 non-*Citrobacter* and *Salmonella* species, including 1 *A. guillouiae*, 2 *E. cloacae*, 6 *K. oxytoca*, 2 *K. pneumoniae*, 3 *M. morganii*, 2 *P. aeruginosa*, and 8 *S. marcescens*, the rate of non-susceptibility to ampicillin (20/21, 95.2%), except 1 *S. marcescens*, was the highest, followed by that to tetracycline (17/22, 72.7%), piperacillin (7/24, 29.2%) in 5 *K. oxytoca* and all 2 *K. pneumoniae*, streptomycin (6/22, 27.3%) in all 6 *K. oxytoca*, imipenem (3/24, 12.5%), cefoxitin (2/21, 9.5%), and chloramphenicol (1/22, 4.5%). The three imipenem-non-susceptible isolates were *M. morganii*, which has intrinsic imipenem resistance. The two cefoxitin-non-susceptible isolates were *E. cloacae*. The one chloramphenicol-non-susceptible isolate was *M. morganii*.

### 3.3. AMR Determinants

We characterized the genotypes of 44 cefoxitin-non-susceptible *Citrobacter* species (37 *C. freundii*, 5 *C. youngae*, and 2 *C. murliniae*) and 2 *E. cloacae* isolates. In addition, we performed genotyping of a chloramphenicol-non-susceptible *M. morganii* isolate. Among them, no plasmid-mediated AmpC β-lactamase (MOX-type, CMY-type, LAT-type, BIL-type, DHA-type, ACC-type, MIR-type, ACT-type, or FOX-type) genes producing *Citrobacter* species and *E. cloacae* were detected, except one AZECL-14, AmpC conserved, producing *E. cloacae* (DCGFes 75 in Table S1). In addition, there was one *M. morganii* isolate (DCGFes 23 in Table S1) harboring the cat gene.

### 3.4. Serological Prevalence of the Salmonella enterica subsp.

All 33 isolates belonged to a single species, *S. enterica* strain. As shown in Table 3, a total of 8 different serotypes were identified among the 33 *S. enterica* subsp. isolates tested in this study. The somatic (O) antigens of the different groups were C (69.7%), D (21.2%), V (6.1%), and M (3.0%), indicating that groups C and D were predominant. For the serological distribution, II 6,7:g,[m],s,t:[z42] and Virginia in group C were the most prevalent (n = 9, 27.3%), followed by Lindenburg in group C and Durban in group D (n = 4, 12.1%), Ouakam in group D (n = 3, 9.1%), Christiansborg in group V (n = 2, 6.1%), and Mbandaka in group C and Pomona in group M (n = 1, 3.0%).

## 4. Discussion

Animals and humans are known to share enteric bacterial species, which means that these species can potentially cause disease in each other [25]. In China, several species of geckos have been used as medicinal ingredients in traditional Chinese medicine [26]. Among reptiles, many reports have documented that various species of common house geckos have a role as reservoirs or carriers in the spread of well-known enteric bacterial zoonotic pathogens [5,6,7,8]. To the best of our knowledge, this study is the first to report on the prevalence and characteristics of zoonotic pathogens, especially Gram-negative bacteria, isolated from fecal samples of crested geckos in South Korea. In this study, for the first time, *A. guillouiae* (n = 1), *M. morganii* (n = 3), and *P. aeruginosa* (n = 2) were identified from fecal samples of crested geckos worldwide. These organisms could be particularly transmitted to young children and elderly people by close contact with the gecko or by handling feces-contaminated environmental stuff. As such, individuals should be careful from the standpoint of hygiene management during breeding and caring for crested geckos, because associated organisms can potentially cause disease.

Different bacterial distributions were identified in terms of the groups, which included groups 1 and 2; these groups were categorized according to diet type in this study (Table S1). *C. freundii* (10/32, 31.3%), *S. marcescens* (8/32, 25.0%), and *C. amalonaticus* (n = 6/32, 18.8%) were the most prevalent strains in crested gecko group 1, which was fed superworms, while *S. enterica* subsp. (32/75, 42.7%) and *C. freundii* (27/75, 36.0%) were dominant strains in crested gecko group 2, which were fed crickets. This study is also the first to report the isolation of *Salmonella* species from the crested gecko in South Korea. Regarding the serological identification of *S. enterica* subsp. clinical isolates in South Korea, serogroups B and C were the most common serogroups, as reported in Kuwait [27]. *S. enterica* subsp. serotype I 4,[5],12:i:- was the main serotype identified, followed by Enteritidis, Bareilly, Typhimurium, and Infantis. These five serotypes accounted for more than 60% (n = 430) of the 669 isolates [28]. In contrast, serogroups C and D were the predominant serological types in this study (Table 3). Only three *S. enterica* subsp. serotypes (II 6,7:g,[m],s,t:[z42], Mbandaka, and Pomona) have been identified among clinical isolates in South Korea [29]. However, the remaining five unusual serotypes (Christiansborg, Durban, Lindenburg, Ouakam, and Virginia) were not previously reported among clinical isolates in South Korea. These differences in *Salmonella* serotypes between crested geckos and humans seem to be due to differences in diet composition, such as the presence of a pathogenic reservoir originating from the cricket, rather than due to contamination by environmental conditions in their living environment. *Salmonella* is one of the most widespread agents that causes gastrointestinal infections in humans and animals. In this regard, there would be no difficulty in treating *Salmonella* infections caused by crested geckos because the 33 *S. enterica* subsp. isolates were susceptible to most antibiotics, except ampicillin and streptomycin. However, the spread of new serotypes that have not been reported among clinical isolates in South Korea should not be overlooked.

Antimicrobial resistance is a current human- and animal-health-threatening issue worldwide [30], indicating that animals and humans share antimicrobial-resistant enteric bacteria. In a clinical field in South Korea, *Citrobacter* species that cause a broad range of infections were recently shown to be highly non-susceptible to ampicillin (100%), cefotaxime (98.8%), ciprofloxacin (90.0%), and tetracycline (47.5%) [31]. However, in this study, the non-susceptibility of *Citrobacter* species isolated from the crested geckos was 80.0% for ampicillin, 0.0% for cefotaxime, 2.0% for ciprofloxacin, and 4.0% for tetracycline, showing completely different results compared with those of above clinical isolates. In addition, although the isolation rate of *Salmonella* species is decreasing in South Korea [32], the rates of resistance to ampicillin, chloramphenicol, cotrimoxazole, cefotaxime, and ciprofloxacin were 32.6%, 12.1%, 8.4%, 8.1%, and 3.0%, respectively, according to a surveillance study of clinical *Salmonella* species in South Korea [28]. These resistance rates were also different from those of most of the *Salmonella* species in this study, which were susceptible to 15 antibiotics. Therefore, the sharing of antimicrobial resistance between crested geckos and humans has not yet progressed, at least for *Citrobacter* species and *Salmonella* species in South Korea.

Most AmpC β-lactamase gene-producing bacteria are resistant to β-lactams [33]. The AmpC β-lactamase genes may be divided into two types: (i) a constitutive chromosomal ampC gene as a natural mechanism, particularly the *Citrobacter* species and *Enterobacter* species; and (ii) chromosomally or plasmid-mediated AmpC β-lactamase derived from the chromosome-borne AmpC β-lactamase of *C. freundii* origin (CIT group; BIL-, partial CMY-, and LAT-type gene); *E. cloacae* origin (EBS group; MIR- and ACT-type or AZECL gene); *M. morganii* origin (DHA group; DHA-type gene); *Hafnia alvei* origin (ACC group; ACC-type gene); *Aeromonas* origin (FOX group; FOX-type gene); or the MOX group, including MOX-type and partial CMY-type genes [19]. In this study, we did not detect the above second type of AmpC β-lactamase in the 44 *Citrobacter* species and 1 *E. cloacae* isolates, except 1 AZECL-14 (accession no. KJ949097) producing *E. cloacae* isolates (DCGFes 75 in Table S1). These results indicate that there was no possibility of horizontal transfer related to gene transfer elements such as transposons, integrons, or plasmids.

Among the 50 *Citrobacter* species (37 *C. freundii*, 6 *C. amalonaticus*, 5 *C. youngae*, 2 *C. murliniae*) in this study, all 6 *C. amalonaticus* isolates were susceptible to all tested antibiotics, including cefoxitin. The strain seems to be associated with clonal spread, not sporadic cases via an unknown route, because the six *C. amalonaticus* isolates had the same antibiotic disk zone diameter.

*M. morganii* isolates are primarily resistant to penicillin, ampicillin, first- and second-generation cephalosporins, erythromycin, tigecycline, and colistin, while they are naturally susceptible to a wide range of antibiotics, including piperacillin, third- and fourth-generation cephalosporins, carbapenems, aztreonam, fluoroquinolones, aminoglycosides, cotrimoxazole, and chloramphenicol [34]. In this study, we found one chloramphenicol-non-susceptible *M. morganii* isolate associated with the production of the *cat* gene, which is responsible for chloramphenicol resistance, with non-susceptibility to ampicillin and tetracycline. Although *M. morganii* infection is a rare clinical occasion, there are reports that it can cause potentially fatal systemic infections, such as urinary tract infections, sepsis, pneumonia, wound infections, musculoskeletal infections, CNS infections, pericarditis, chorioamnionitis, endophthalmitis, empyema, and spontaneous bacterial peritonitis [35]. In this regard, in the case of infection with the *M. morganii* isolates identified in this study, there would be no difficulty in treatment because they were susceptible to most of the remaining antibiotics, except for tetracycline and chloramphenicol.

In this study, our results have a limitation. The prevalence and characteristics of enteric bacteria collected from the crested gecko do not represent the distribution and antimicrobial resistance pattern of enteric bacteria isolated from the crested gecko in South Korea because this screening was performed on enteric bacteria collected from the fecal samples of crested geckos bred and cared for by a private keeper in a single household. However, as private keepers breeding reptiles have recently increased, our surveillance for the prevalence and characteristics of *S*. *enterica* subsp., including novel serotypes, in this study is considered to be a meaningful result, especially in the one-health concept.

## 5. Conclusions

Our results show the prevalence and characteristics of enteric bacteria in fecal samples collected from crested geckos in South Korea. The antimicrobial resistance phenotypes of these strains differed from those of clinical isolates. In particular, five of the eight identified *Salmonella* serotypes have not been previously reported among clinical isolates in South Korea. Our results reveal that the transfer of enteric bacteria between crested geckos and humans has not yet progressed, at least in South Korea. However, private keepers breeding the crested gecko are encouraged to practice personal hygiene because the spread of enteric bacteria that have not been reported in clinical isolates should not be overlooked.

## Figures and Tables

**Table 1:** List of primers used in this study.

Primer Name	Target Gene	Nucleotide Sequences (5′ to 3′)	Amplicon Size	References
MOXM-F	MOX, CMY-1-type	GCTGCTCAAGGAGCACAGGAT	520 bp	[23]
MOXM-R		CACATTGACATAGGTGTGGTGC		
CITM-F	LAT, CMY-2-type, BIL	TGGCCAGAACTGACAGGCAAA	462 bp	
CITM-R		TTTCTCCTGAACGTGGCTGGC		
DHAM-F	DHA	AACTTTCACAGGTGTGCTGGGT	405 bp	
DHAM-R		CCGTACGCATACTGGCTTTGC		
ACCM-F	ACC	AACAGCCTCAGCAGCCGGTTA	346 bp	
ACCM-R		TTCGCCGCAATCATCCCTAGC		
EBCM-F	MIR, ACT, AZECL	TCGGTAAAGCCGATGTTGCGG	302 bp	
EBCM-R		CTTCCACTGCGGCTGCCAGTT		
FOXM-F	FOX	AACATGGGGTATCAGGGAGATG	190 bp	
FOXM-R		CAAAGCGCGTAACCGGATTGG		
cat-F1	CAT	TTTGAACCAACAAACGACTTT	573 bp	[24]
cat-R1		GGCCTATCTGACAATTCCTGA		
cat-F2		CCAACAAAACGACTTTTAGTATAACC	529 bp	
cat-R2		TCCTGCATGATAACCATCAC		

**Table 2:** Characteristics of the 33 *Salmonella* species isolated from domesticated crested geckos.

No. of the Crested Gecko	Diet Type	Identification	Phenotypic Non-Susceptible	O-Group	Serotype
13	Superworm	*S. enterica*	Amp	C1	II 6,7:g,[m],s,t:[z42]
21	Cricket	*S. enterica*	-	C1	II 6,7:g,[m],s,t:[z42]
23		*S. enterica*	-	D1	Durban
24		*S. enterica*	Amp, S	C1	Mbandaka
25		*S. enterica*	-	C1	II 6,7:g,[m],s,t:[z42]
26		*S. enterica*	S	C1	II 6,7:g,[m],s,t:[z42]
27		*S. enterica*	-	C2	Virginia
28		*S. enterica*	-	D1	Durban
31		*S. enterica*	S	C1	II 6,7:g,[m],s,t:[z42]
32		*S. enterica*	-	C1	II 6,7:g,[m],s,t:[z42]
33		*S. enterica*	-	C2	Virginia
34		*S. enterica*	-	V	Christiansborg
35		*S. enterica*	-	C1	II 6,7:g,[m],s,t:[z42]
36		*S. enterica*	-	V	Christiansborg
40		*S. enterica*	-	C1	II 6,7:g,[m],s,t:[z42]
41		*S. enterica*	-	C2	Virginia
42		*S. enterica*	-	C2	Lindenburg
44		*S. enterica*	-	C2	Virginia
45		*S. enterica*	-	C2	Virginia
46		*S. enterica*	S	D1	Ouakam
47		*S. enterica*	-	C2	Virginia
48		*S. enterica*	-	C2	Lindenburg
52		*S. enterica*	-	C2	Lindenburg
53		*S. enterica*	S	D1	Durban
54		*S. enterica*	-	M	Pomona
55		*S. enterica*	S	D1	Ouakam
57		*S. enterica*	S	D1	Durban
60		*S. enterica*	Amp	C2	Lindenburg
64		*S. enterica*	-	C2	Virginia
65		*S. enterica*	-	C2	Virginia
68		*S. enterica*	-	C2	Virginia
71		*S. enterica*	S	D1	Ouakam
76		*S. enterica*	S	C1	II 6,7:g,[m],s,t:[z42]

**Table 3:** Somatic (O) antigen group and serotype distribution of *Salmonella enterica* subsp., isolates.

Serogroup	Group C		Group D		Group M		Group V	
	Serotype (H Antigen)	No. (%)	Serotype (H Antigen)	No. (%)	Serotype (H Antigen)	No. (%)	Serotype (H Antigen)	No. (%)
Serotype	II 6,7:g,[m],s,t:[z42] (g,m,s,t)	9 (27.3)	Durban (a,e,n,z15)	4 (12.1)	Pomona (y,1,7)	1 (3.0)	Christiansborg (z4,z24)	2 (6.1)
	Virginia (d,1,2)	9 (27.3)	Ouakam (g,m)	3 (9.1)				
	Lindenburg (i,1,2)	4 (12.1)						
	Mbandaka (z10,e,n,z15)	1 (3.0)						
Total (n = 33)	23 (69.7)		7 (21.2)		1 (3.0)		2 (6.1)	

## Data Availability

Data are contained within the article.

## Supplementary Materials

The following supporting information can be downloaded at https://www.mdpi.com/article/10.3390/life15030405/s1, Table S1: List of 107 Gram-negative isolates collected from 76 domesticated crested geckos.
