# Supplementary material for: Serological Distribution of Salmonella enterica subsp. Isolated from Feces of Domesticated Crested Gecko (Correlophus ciliates) in Busan Province, South Korea
Source: Life (Basel). 2025 Mar 5;15(3):405. doi: 10.3390/life15030405 (PMC11944151; doi:10.3390/life15030405)
Supplement: Supplementary file 1 [file life-15-00405-s001.zip › life-3438300-supplementary.pdf]

Table S1 List of 107 gram-negative isolates collected from 76 domesticated crested geckos

Strain number of fecal samples of each crested gecko	Groups for diet types	No. of isolates	Gram-negative species
DCGFes1	group 1 fed on superfood	0	-
DCGFes 2		0	-
DCGFes 3		2	*Citrobacter amalonaticus*, *Serratia marcescens*
DCGFes 4		2	*C. amalonaticus*, *S. marcescens*
DCGFes 5		4	*C. amalonaticus*, *Citrobacter freundii*, *S. marcescens*, *Klebsiella oxytoca*
DCGFes 6		1	*S. marcescens*
DCGFes 7		1	*Pseudomonas aeruginosa*
DCGFes 8		0	-
DCGFes 9		1	*S. marcescens*
DCGFes 10		1	*C. freundii*
DCGFes 11		3	*C. freundii*, *Morganella morganii*, *S. marcescens*
DCGFes 12		2	*Acinetobacter guillouiae*, *C. freundii*
DCGFes 13		5	*C. fruendii*, *Citrobacter murliniae*, *Citrobacter youngae*, *Salmonella* spp., *S. marcescens*
DCGFes 14		1	*C. freundii*
DCGFes 15		1	*C. amalonaticus*
DCGFes 16		3	*C. amalonaticus*, *C. freundii*, *S. marcescens*
DCGFes 17		0	-
DCGFes 18		2	*C. amalonaticus*, *C. freundii*
DCGFes 19		1	*C. freundii*
DCGFes 20		2	*C. freundii*, *C. murliniae*
DCGFes 21	group 2 fed on infect (Cricket)	1	*Salmonella* spp.
DCGFes 22		0	-
DCGFes 23		2	*M. morganii*, *Salmonella* spp.
DCGFes 24		2	*C. freundii*, *Salmonella* spp.
DCGFes 25		2	*C. freundii*, *Salmonella* spp.
DCGFes 26		2	*C. freundii*, *Salmonella* spp.
DCGFes 27		2	*C. freundii*, *Salmonella* spp.
DCGFes 28		2	*C. freundii*, *Salmonella* spp.
DCGFes 29		2	*C. freundii*, *M. morganii*
DCGFes 30		0	-
DCGFes 31		1	*Salmonella* spp.
DCGFes 32		1	*Salmonella* spp.
DCGFes 33		2	*C. freundii*, *Salmonella* spp.
DCGFes 34		3	*C. freundii*, *C. youngae*, *Salmonella* spp.
DCGFes 35		2	*C. freundii*, *Salmonella* spp.
DCGFes 36		3	*C. freundii*, *K. oxytoca*, *Salmonella* spp.

DCGFes 37	0	-
DCGFes 38	0	-
DCGFes 39	0	-
DCGFes 40	2	*C. freundii*, *Salmonella* spp.
DCGFes 41	3	*C. freundii*, *Klebsiella pneumoniae*, *Salmonella* spp.
DCGFes 42	2	*C. freundii*, *Salmonella* spp.
DCGFes 43	1	*C. freundii*
DCGFes 44	2	*K. pneumoniae*, *Salmonella* spp.
DCGFes 45	2	*C. freundii*, *Salmonella* spp.
DCGFes 46	3	*C. freundii*, *C. youngae*, *Salmonella* spp.
DCGFes 47	2	*C. freundii*, *Salmonella* spp.
DCGFes 48	3	*C. freundii*, *K. oxytoca*, *Salmonella* spp.
DCGFes 49	2	*C. freundii*, *K. oxytoca*
DCGFes 50	2	*C. youngae*, *P. aeruginosa*
DCGFes 51	0	-
DCGFes 52	2	*K. oxytoca*, *Salmonella* spp.
DCGFes 53	2	*C. freundii*, *Salmonella* spp.
DCGFes 54	2	*C. youngae*, *Salmonella* spp.
DCGFes 55	2	*C. freundii*, *Salmonella* spp.
DCGFes 56	0	-
DCGFes 57	2	*C. freundii*, *Salmonella* spp.
DCGFes 58	0	-
DCGFes 59	0	-
DCGFes 60	2	*C. freundii*, *Salmonella* spp.
DCGFes 61	0	-
DCGFes 62	0	-
DCGFes 63	1	*C. freundii*
DCGFes 64	1	*Salmonella* spp.
DCGFes 65	1	*Salmonella* spp.
DCGFes 66	1	*C. freundii*
DCGFes 67	0	-
DCGFes 68	2	*C. freundii*, *Salmonella* spp.
DCGFes 69	1	*Enterobacter cloacae*
DCGFes 70	0	-
DCGFes 71	2	*C. freundii*, *Salmonella* spp.
DCGFes 72	1	*K. oxytoca*
DCGFes 73	0	-
DCGFes 74	0	-
DCGFes 75	1	*E. cloacae*
DCGFes 76	1	*Salmonella* spp.

Abbreviation: DCGFes, domesticated crested gecko fecal sample
